# Finding a calling, not a job: How an East Tennessee girl transformed aging and public health

**DOI:** 10.3389/fpubh.2022.922526

**Published:** 2022-08-04

**Authors:** Omolola E. Adepoju

**Affiliations:** Department of Health Systems and Population Heath Sciences, University of Houston College of Medicine, Houston, TX, United States

**Keywords:** aging, mentor—mentee communication, public health, women, science—general

Catherine Hawes, Ph.D., to me, is one of the very best role models in the aging and public health sector. Born in East Tennessee and raised conservative, Catherine proved to be a non-conformist in her beliefs, challenging norms that tended to relegate women and persons of color to second-class citizens. For women, completing a bachelor's degree was a huge accomplishment in the 1960s, and Catherine went even further to complete a doctoral program in American Government and Politics. Her lived experiences regarding fairness and gender equality, as well as caring for her sick mother contributed to Catherine's career path—which later became a calling. In an era preceding the current “wokeness” and social awareness of racism, Catherine was a trailblazer in treating everyone fairly, regardless of what or how they identified. When it comes to issues related to aging and public health, there are no ifs, ands, or buts—Catherine's mission is health equity for all older adults. Her passion for aging and public health is infectious, and as I find myself doing more work along this line, I cannot think of anyone more deserving or “sufficiently brilliant^1^” to write about.

I met Catherine in August 2009, when started a PhD program in Health Services Research at Texas A&M University. She taught health policy in a manner that made one never want the class to end—something I cannot say about other courses taught by other professors. Monday afternoons was like sitting with a former President and learning the inner working of how health policy is made, the role of interest groups, and how incrementalism over the years had shaped our current healthcare delivery system. Since this period was before the passage of the Affordable Care Act (ACA), we had many conversations on how the Obama administration should shape healthcare health care reform. Including examples from her time working on the Hill to consulting with numerous leaders in Congress, state health departments, and national think tanks, Catherine's class was a must for all health services researchers-in-training. As an immigrant, I found it surprising that the U.S. Health care system that I came to the U.S. to learn about, although much better that in my home country of Nigeria, was far from perfect—and without a quick fix.

We discussed comparative health systems and how learnings from other countries, such as medical homes and accountable care, could inform health care reform strategies under consideration by the Obama administration. By the end of the semester, I wanted to work with this intellectual giant and mentor to many researchers in aging and public health. I asked Catherine about working as a research assistant with her and her husband, Charles, who was also at Texas A&M. Together, their role in positively impacting scholars kindles inspiration and propensities to pursue performance excellence.

In addition to her wealth of knowledge, Catherine is hardworking and graceful, always willing to engage in civil discourse on all things health policy. We have often disagreed on political stance, but her ability to promote judgment-free dialogues has endeared her to many. Not surprisingly, for almost 40 years (1976–2013), Catherine was active in research, teaching, and health policymaking, with an emphasis on defining, measuring, and assuring quality in long-term care. She led several projects on assisted living and residential care, including quality measurement and improvement in residential care and assisted living for the Agency for Healthcare Research and Quality. Her landmark study on the effect of regulation on the quality of care in board and care homes recognized licensures alone as insufficient in ensuring that homes provide care above a threshold of minimum performance (board and care homes provide supervised living environments in the absence of family support and serve as an alternative to nursing home placement) (1). This work resulted in significant policy changes, with an emphasis on federal oversight of nursing home quality. In addition, Catherine served on a number of national advisory committees, including the Institute of Medicine's (IOM) Committee on Nursing Home Regulation. She has also provided papers and testimony to other IOM committees on quality assurance in Medicare, improving quality in long-term care, and preventing elder abuse in residential long-term care settings. She currently serves on the National Policy Council of AARP, which makes recommendations on public policy to the AARP Board of Directors.

Perhaps her most visible contribution is Catherine's role in the development of the Minimum Dataset for Nursing Home Resident Assessment and Care Screening (MDS), the holy grail of clinical assessments in long-term care facilities. The MDS is used to screen and assess physical, psychological and psycho-social functioning, providing a multidimensional view of a resident's functional status (2). Anyone who has had a parent, grandparent, aunt, uncle, friend, or child in a nursing home has benefited from Catherine's work on the MDS. This comprehensive assessment is conducted annually for all long-term care residents, regardless of payer type. Over the past 20 years, the MDS has played a vital role in the Medicare and Medicaid reimbursement system and in monitoring the quality of care provided to nursing facility residents. From a scholarly perspective, this work (3) has been cited almost 1,000 times.

To the best of my knowledge, Catherine has mentored more than 100 health services researchers currently studying aging and public health across diverse settings, including universities, health departments, Congress, and non-profit think tanks. Like me, many of her mentees have continued work in aging and public health, focusing on shifts in the demographic dividend, care for the aging boomers (who now live longer than their parents), mental health for older adults, and going beyond measurements to address barriers that promote racial and ethnic disparities in health.

## Author contributions

OA conceptualized and wrote the manuscript.

## Conflict of interest

The author declares that the research was conducted in the absence of any commercial or financial relationships that could be construed as a potential conflict of interest.

## Publisher's note

All claims expressed in this article are solely those of the authors and do not necessarily represent those of their affiliated organizations, or those of the publisher, the editors and the reviewers. Any product that may be evaluated in this article, or claim that may be made by its manufacturer, is not guaranteed or endorsed by the publisher.

## Footnotes

^1^During a conversation with Catherine Hawes, on June 28, 2022, she recounted how a male colleague once questioned whether females and racialized groups were sufficiently brilliant to become faculty members.
